# Tissue-specific patterns of gene expression in the epithelium and stroma of normal colon in healthy individuals in an aspirin intervention trial

**DOI:** 10.1016/j.gdata.2015.08.029

**Published:** 2015-09-01

**Authors:** Sushma S. Thomas, Karen W. Makar, Lin Li, Yingye Zheng, Peiying Yang, Lisa Levy, Rebecca Y. Rudolph, Paul D. Lampe, Min Yan, Sanford D. Markowitz, Jeannette Bigler, Johanna W. Lampe, John D. Potter

**Affiliations:** a Fred Hutchinson Cancer Research Center, Seattle, WA 98109, USA; b M.D. Anderson Cancer Center, Houston, TX 77030, USA; c Case Western Reserve University School of Medicine, Cincinnati, OH 44106, USA; d Amgen Corporation, Seattle, WA 98119, USA

## Abstract

Regular aspirin use reduces colon adenoma and carcinoma incidence. UDP-glucuronosyltransferases (UGT) are involved in aspirin metabolism and clearance, and variant alleles in UGT1A6 have been shown to alter salicylic acid metabolism and risk of colon neoplasia. In a randomized, cross-over, placebo-controlled trial of 44 healthy men and women, homozygous for UGT1A6*1 or UGT1A6*2, we explored differences between global epithelial and stromal expression, using Affymetrix U133 + 2.0 microarrays and tested effects of 60-day aspirin supplementation (325 mg/d) on epithelial and stromal gene expression and colon prostaglandin E2 (PGE2) levels. We conducted a comprehensive study of differential gene expression between normal human colonic epithelium and stroma from healthy individuals. Although no statistically significant differences in gene expression were observed in response to aspirin or UGT1A6 genotype, we have identified the genes uniquely and reproducibly expressed in each tissue type and have analyzed the biologic processes they represent. Here we describe in detail how the data, deposited in the Gene Expression Omnibus (GEO) – accession number GSE71571 – was generated including the basic analysis as contained in the manuscript published in BMC Medical Genetics with the PMID 25927723 (Thomas et al., 2015 [9]).

**Table:** 

Specifications	
Organism/cell line/tissue	*Homo sapiens*/colon/epithelial and stromal tissue
Sex	Males and females
Sequencer or array type	Affymetrix GeneChip HGU133 + 2.0 microarrays
Data format	Raw and normalized
Experimental factors	We tested effects of 60-day aspirin supplementation (325 mg/d) on epithelial and stromal gene expression in 44 healthy men and women who were randomly assigned, blocked on sex and genotype, to the order in which they received treatment A or treatment B. (Note: The type of treatment i.e., pill type active or placebo was masked).
Experimental features	The washout period between intervention periods was three months. A sigmoidoscopy was performed at least 60 days after day 1 of each intervention period. Therefore, two biopsies were obtained from each participant and then processed, where by, epithelial and stromal fractions were separated and analyzed using Affymetrix U133 + 2.0 microarrays. This resulted in obtaining total RNA from 88 epithelial and 88 matched stromal samples. A total of 176 gene arrays were run.
Consent	All study procedures and materials were approved by the Fred Hutchinson Cancer Research Center Human Research Protection Program, Institutional Review Board Committee C and informed, written consent was obtained from all participants prior to their starting the study.
Sample source location	Study participants were recruited from the greater Seattle, WA area between June 2003 and March 2007

## 1 Direct link to deposited data

The direct link to deposited files is at http://datalink.elsevier.com/midas/datalink/api/downloadfiles?items=17107-17108-17109.

The direct link to deposited genomic data is at http://www.ncbi.nlm.nih.gov/geo/query/acc.cgi?acc=GSE71571.

## 2 Experimental design, materials and methods

The objectives of this study were to measure effects of an aspirin intervention on gene expression in normal colonic epithelial and stromal tissue in healthy humans and to determine whether response differed by UGT1A6*2 genotype. We also sought to characterize gene expression differences within colonic tissue microenvironments by identifying genes that were differentially expressed between epithelial and stromal tissue.

### 2.1 Participants

**Fig. 1:**
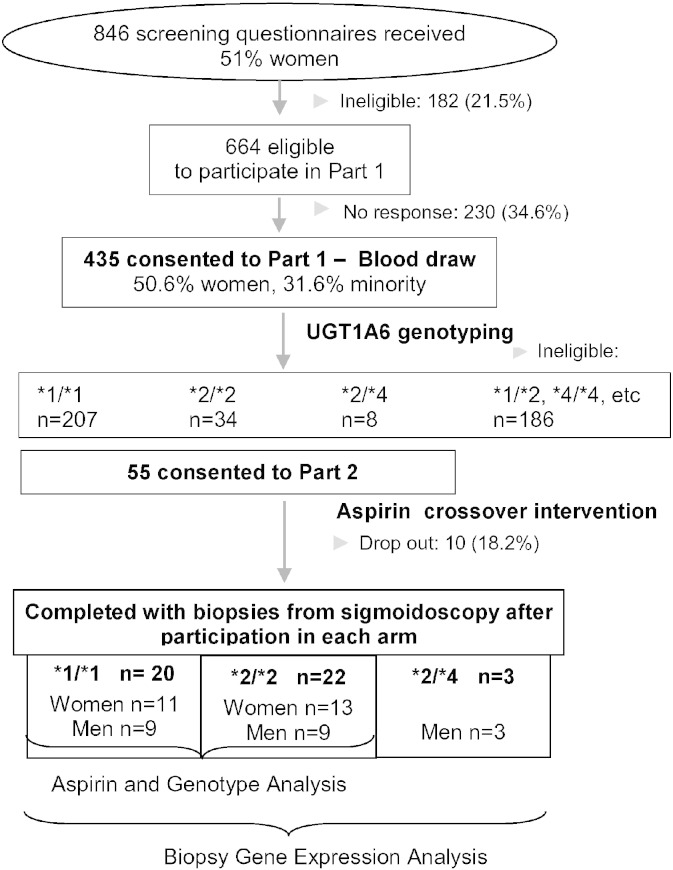
Flow chart of participant enrollment and study design.

We recruited healthy men and women, ages 20 to 45 years, from the greater Seattle area between June 2003 and March 2007. Participants were recruited from among those who completed a cross-sectional study of diet and aspirin metabolism (Fig. 1). Potential eligibility was assessed by questionnaire. Exclusion criteria included tobacco use, consumption of > 2 alcoholic beverages/day (equivalent to 720 ml beer, 240 ml wine, 90 ml hard liquor), regular use of prescription or over-the-counter medications, known intolerance of aspirin or other non-steroidal anti-inflammatory drugs (NSAID), weight loss or gain of > 4.5 kg in the past year, current or planned pregnancy, breastfeeding, bleeding disorder, anemia, renal insufficiency, hepatic dysfunction (e.g., cirrhosis, hepatitis, abnormal liver function tests), chronic lung disease, hypertension, congestive heart failure, angina, recent myocardial infarction, history of endocarditis, aortic or iliac aneurysm, history of stroke or transient ischemic attack, diabetes, recent pelvic surgery, history of gastrointestinal disorder (e.g., gastric or duodenal ulcer, ulcerative colitis, Crohn's disease, celiac sprue, HNPCC, familial adenomatous polyposis, pancreatic disease, previous gastrointestinal resection, radiation or chemotherapy) and cancer (other than non-melanoma skin cancer).

As part of the cross-sectional study, participants completed a self-administered food frequency questionnaire and a health and demographic survey, and provided a fasting morning blood that was used for UGT1A6 genotyping [1]. We determined the UGT1A6*2 genotype of 434 participants by Sanger sequencing of a 268 bp fragment of exon 1, amplified by PCR as described previously [2].

### 2.2 Study design

The study was a randomized, double-blind crossover clinical trial designed to examine the effects of aspirin on gene expression in colonic mucosa. Our goal was to have at least 40 participants complete both arms of the trial. Participants were selected on the basis of their genotype of rs2070959 (T181A) and rs1105879 (R184S) — 20 with the UGT1A6 *1/*1 genotype (homozygous for the major allele at both SNPs) and 20 with the UGT1A6 *2/*2 genotype (homozygous for the minor allele at both SNPs). All participants with a *2/*2 genotype and sex-matched participants with a *1/*1 genotype were invited to consider participation in the trial. Fifty-five individuals (23 men), mean (SD) age 30.1 (6.5) years, were randomized. Ten participants discontinued the study: 3 did not tolerate the Fleets Phospho-soda preparation prior to the first sigmoidoscopy, 2 started on prescription medications, 2 refused sigmoidoscopy, 3 left for reasons unrelated to the study and sufficient total RNA could not be extracted from one participant. Forty-two participants were included in the analysis of intervention and UGT1A6 genotype effects and an additional 3 participants with the UGT1A6 *2/*4 genotype were randomized and included in the expression array analysis of stromal and epithelial tissue. (Initially, we randomized *2/*4 individuals into the study because the phenotype was thought to be similar to that of *2/*2; however, with the emergence of new data [10], we decided that the 2 genotypes may be sufficiently different and therefore excluded *2/*4 individuals from the intervention analysis.)

To ensure that participants could take aspirin safely for 60 days and tolerate sigmoidoscopy, each participant underwent a clinical assessment before entering the study that included a detailed medical history, measurement of blood pressure and complete blood count, liver panel, chemistry panel, blood urea nitrogen, serum creatinine, urinalysis, and in women only, a pregnancy test. In addition, a physician interviewed and examined participants before each of their sigmoidoscopies. The laboratory assays to assess the health of participants were completed by a commercial lab (CLIA licensed Quest Diagnostics, Seattle, WA).

Because this was a randomized crossover trial, each person served as his/her own control, receiving both intervention (aspirin) and control (placebo) at 2 different periods, with a washout period between periods. Participants were randomly assigned, blocked on sex and genotype, to the order in which they received aspirin or placebo. Eligible participants took 325 mg aspirin or a visually identical placebo by mouth daily, for 60 days. The washout period between intervention periods was 3 months. A sigmoidoscopy was performed at least 60 days after day 1 of each intervention period. Participants took the study medication up until 24 h before their sigmoidoscopies. During each treatment period, we monitored adherence to the study medication by pill count.

### 2.3 Sigmoidoscopy, biopsy collection, and tissue separation

Participants prepared for sigmoidoscopy by following standard instructions for use of Fleet Phospho-soda Oral Saline Laxative (Lynchburg, VA), which included adherence to a clear-liquid diet for 24 h before the procedure. At each sigmoidoscopy, we used large-cup flexible biopsy forceps (Precisor Disposable, Bard, Billerica, MA) to obtain biopsies of normal-appearing mucosa from the sigmoid colon. Forty-five participants completed both sigmoidoscopies. Within 1 min of removal from the colon, three biopsies were transferred to Hank's buffer containing 20 mM EDTA and 40 mM dithiothreitol at 4 °C and were held on ice for 10–15 min. The epithelial cells were separated from the stromal layer by vortexing [3]. The stromal layer was removed with an autoclaved toothpick and epithelial fraction was then collected by centrifugation. Both fractions were frozen and stored in liquid nitrogen until RNA extraction.

### 2.4 RNA extraction and microarray procedures

Participants were labeled consecutively. There are 4 samples from each person designated A1–A4 (participant 1); A5–A6 (participant 2); B1–B4 (participant 3) etc. to W1–W4 (participant 44). Within the data for each person, odd numbers (1 and 3) are epithelial tissue and even numbers (2 and 4) are stromal tissue. The epithelial and stroma from each intervention period are paired together (e.g., A1 and A2 are from the first treatment Participant 1 received; A3 and A4 are from the second treatment participant 1 received). Each participant's epithelial and stromal samples were processed at the same time. Total RNA was extracted with RNeasy extraction kits (Qiagen, Valencia, California) and treated with DNAse I according to the manufacturer's protocol. The integrity of total RNA samples was verified by visual analysis of 18S and 28S bands on an ethidium bromide-stained 1.5% agarose gel. RNA was quantified using the Quant-iT RiboGreen RNA assay Kit (Molecular Probes, Eugene, Oregon). We obtained sufficient RNA from 44 of the 45 participants. This resulted in obtaining total RNA from 88 epithelial samples and 88 matched stromal samples. cRNA was synthesized from 100 ng of total RNA, biotin-labeled by the GeneChip Two-cycle cDNA synthesis labeling protocol (Affymetrix, Inc., Santa Clara, California), and then hybridized to HGU133 plus 2.0 expression arrays. GeneChips were washed and stained in the Affymetrix Fluidics Station 450. GeneChips were scanned using the GeneChip Scanner 3000 7G. Each microarray contained 54,675 probes representing 38,500 genes. The quality control (QC) criteria used to assess sample and chip performance were fold-amplification after two cycles of in vitro transcription (to be at least 40-fold), and increasing signal strengths and ‘present’ calls for poly-A RNA spike-in controls (lys, phe, thr, dap) and hybridization controls (bioB, bioC, bioD and cre). Microarray data are publicly available in Gene Expression Omnibus (GEO) with the accession number GSE71571.

### 2.5 Real-time quantitative reverse transcription-polymerase chain reaction (qRT-PCR)

To validate the microarray data, TaqMan RT-PCR was performed on a total of 20 RNA samples, which included both pairs of stromal and epithelial samples from 10 of the 44 individuals. To ensure an RT performance of equal quality, all samples were reverse transcribed simultaneously by a two-step reverse transcription with SuperScript RT II (Invitrogen, Carlsbad, California). Relative gene expression was examined by TaqMan PCR using 2 × TaqMan Gene Expression Master Mix (Applied Biosystems, Foster City, California) and TaqMan Gene Expression Assays for two epithelial genes (ABCC3, MLPH; assay identifiers Hs00358677_m1 and Hs00983106_m1) and two stromal genes (ITGα8 and ANXA1; assay identifiers Hs00943530_m1 and Hs00945401_m1). Human β-glucuronidase (GUSB) was used as the normalization control based on its consistent expression across all samples in the microarray analysis. Relative quantification was performed with the ABI Prism 7900 HT sequence detection system and calculated by the comparative CT method.

### 2.6 PGE2 analysis

PGE2 in whole biopsies collected at the end of each treatment period was measured with enzyme immunoassay kits from Assay Design (Farmingdale, NY). Briefly, each frozen biopsy tissue sample was transferred to a sealed microcentrifuge tube to which 500 μl of ice-cold tissue homogenization buffer had been added previously [4]. Each sample was homogenized by an Ultrasonic Processor (Misonix, Farmingdale, NJ) at 4 °C for 3.5 min × 2 with a 1-min rest in between and then centrifuged at 16,000 rpm for 5 min at 4 °C. An aliquot (100 μl) was mixed with 100 μl of assay buffer and acidified with 20 μl 1 N citric acid. The acidified solution was then applied to a Sep.-Pak C18 cartridge (Waters Corp., Milford, MA) that had been preconditioned with methanol and water. Prostaglandins were eluted with 2 ml of hexane:ethyl acetate (1:1). The eluate was evaporated under a stream of nitrogen, and the residue was dissolved in 25 μl ethanol and 200 μl assay buffer. PGE2 was then measured with ELISA kits according to the manufacturer's instructions. Protein levels were determined by a Bradford protein assay (Bio-Rad, Hercules, CA). Levels of PGE2 were normalized to protein concentrations and expressed as ng/mg protein.

### 2.7 Data analysis–microarray analysis

Data were normalized by the Robust Multi-array Analysis with correction for GC content of the oligo (GC-RMA) [5]. Subsequently, the probe-set expression levels were log2-transformed using GC-RMA (version 2.40.0) and R version 3.2.1 (2015–06–18) with default function settings. Nonspecific filtering was done where estimated intensities were required to be above 100 fluorescence units in at least 25% of the samples and the interquartile range (IQR) across all of the samples on the log base 2 scale was expected to be at least 0.6. A total of 6227 genes passed nonspecific filtering. Differential expression between tissue types in biopsies collected at the end of each treatment period was determined by using a paired t-test. The false discovery rate (FDR) was calculated using the procedure proposed by Benjamini and Hochberg [6]. Visualization of the statistically significant genes was performed in GeneSpring 7.0 with per chip and per gene normalizations (Agilent Technologies, Wilmington, Delaware). Ingenuity Pathways Analysis (IPA, Release Summer 2013) (Ingenuity Systems, Redwood City, California, www.ingenuity.com) was used to determine the relevant functions and pathways expressed in each tissue type. A list of the top genes in each pathway was identified using GeneSpring and heat maps for each pathway were generated by clustering these genes in Gene Cluster 3.0 [7] and then visualizing the clusters using Java TreeView [8]. Differential expression between interventions within each tissue type and between two UGT1A6 genotypes (*1/*1 and *2/*2) were also examined by using the same paired t-test and the generalized estimating equation approach. Genes that were statistically significantly differentially expressed between stroma and epithelium at the end of both treatment periods were included in further pathway analysis.

### 2.8 Data analysis–PGE2 analysis

Because the distributions of PGE2 levels measured in biopsies collected at the end of each treatment period were skewed, the data were logarithmically transformed. The difference between treatments was determined by using paired t-test and a random-effects model that takes into account the crossover design. The comparison between aspirin and placebo was further examined after stratification for sex, age, and UGT1A6 genotypes.

## 3 Results

Tissue-specific patterns of gene expression in epithelium and stroma of normal human colon. From 38,500 genes analyzed by microarray, we identified genes that were statistically significantly differentially expressed between epithelium and stroma in each treatment period. Because there were no intervention differences, we combined the observations and restricted subsequent analyses to those genes that were differentially expressed in both treatment periods (P < 0.01, FDR < 0.001). This resulted in further exploration of 4916 genes. Of these, 2088 were higher in stroma and 2828 were higher in epithelium.

**Fig. 2:**
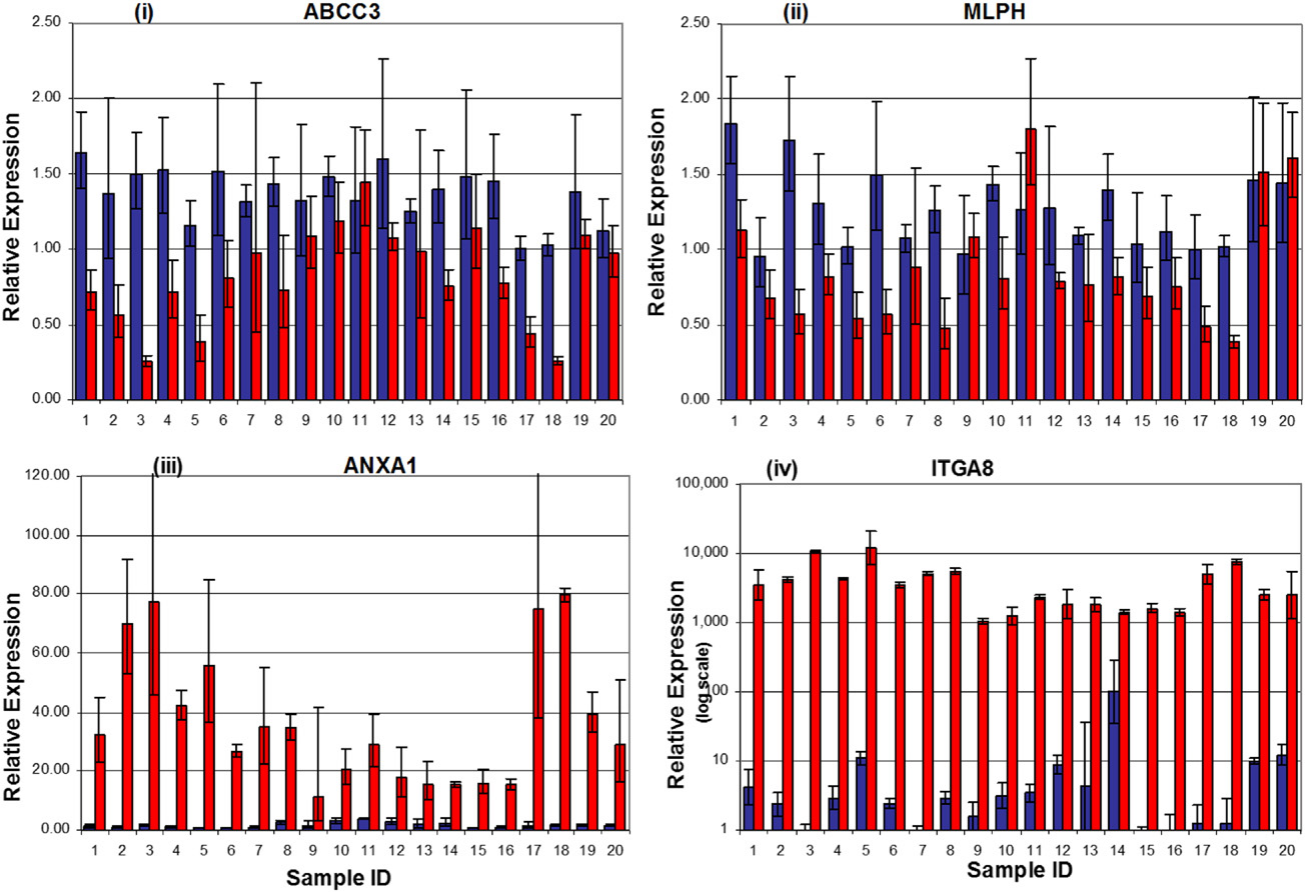
Real-time PCR confirms differences in gene expression in colonic epithelium and stroma. Quantitative real-time RT-PCR was done on both biopsies (taken at two separate visits) from 10 study participants for four candidate genes. The histogram bars from the same individual are located next to each other, i.e., samples 1 and 2 are from the same person but taken at two separate visits. Total RNA was reverse transcribed and then amplified by PCR and normalized to β-glucuronidase (GUSB) expression. Blue bars: epithelial tissue. Red bars: stromal tissue. Upper panel shows expression of: (i) ABCC3, an epithelial gene involved in drug and lipid metabolism; and (ii) MLPH (melanophilin), an epithelial gene involved in protein binding and protein transport. Lower panel shows expression of: (iii) ANXA1 (annexin A1), a stromal gene involved in cell migration and cell adhesion; and (iv) ITGA8 (integrin α8), a stromal gene involved in extracellular matrix formation.

Stromal and epithelial patterns of expression observed in the microarray data were confirmed by qRT-PCR of selected genes (Fig. 2). Expression of the selected epithelial-specific genes, ABCC3 and MLPH1, was detectable in the stromal samples, but at a lower level than in the epithelial samples. In contrast, the selected stromal-specific genes, ANXA1 and ITGA8, generally lacked detectable expression in the epithelial samples.

To understand the biologic processes represented by differentially expressed genes, we analyzed those showing statistically significant differences with Ingenuity Pathways Analysis (IPA). Although both compartments expressed genes involved with cancer, cell death, cell signaling and cellular movement, several pathways and biologic functions appeared to be unique to one or the other tissue. Table 1 lists the major differentially expressed pathways and functions by tissue type.

## 4 Conclusion

We have identified the genes uniquely and reproducibly expressed in each tissue type and have analyzed the biologic processes they represent. The wide differences in expression between stroma and epithelium suggest that future study of gene expression in colonic epithelium may benefit from separation of the tissue so as to focus the analysis on a specific tissue type.

## Figures and Tables

**Table 1:** Statistically significant pathways and functions in colonic epithelium and stroma.

Epithelial genes	
Canonical pathways	p-Value
Xenobiotic metabolism signaling	0.000124
Fatty acid metabolism	0.00262
*Apoptosis signaling*	0.22
Biological function	
Epithelial genes p-value	6.25E-07-2.47E-02
Small molecule biochemistry	6.25E-07-2.47E-02
Molecular transport	5.89E-05-2.47E-02
*Carbohydrate metabolism*	7.48E-04-2.47E-02
*Drug metabolism*	1.80E-03-2.47E-02
*Protein trafficking*	2.34E-03-2.47E-02
*Nucleic acid metabolism*	8.58E-03-2.47E-02
*Amino acid metabolism*	1.23E-02-2.47E-02

Stromal genes	

Canonical pathways	p-Value
*Complement system*	2.49E-08
*Antigen presentation pathway*	0.00000018
IL-4 signaling	0.0000656
Biological function	
Inflammation	8.74E-28-8.17E-06
Cell-to-cell signaling	1.90E-27-1.09E-05
*Immunological disease*	5.34E-25-4.44E-06
Hematological system development & function	4.50E-19-1.12E-05
Immune response	4.50E-19-1.12E-05

Ingenuity Pathway Analysis of selected biological functional groups and metabolic and signaling pathways for all statistically significant genes of both tissue types. Range of p-values indicates the significance values of the specific sub-functions associated with that particular high-level function. *Italic font indicates that function was unique to that particular tissue type and was essentially not expressed in the other tissue*.
